# Randomized controlled trial comparing letrozole with laparoscopic ovarian drilling in women with clomiphene citrate-resistant polycystic ovary syndrome

**DOI:** 10.3892/etm.2015.2690

**Published:** 2015-08-19

**Authors:** WEI LIU, SHENGNAN DONG, YUMEI LI, LIHONG SHI, WEI ZHOU, YINGLING LIU, JIE LIU, YAZHONG JI

**Affiliations:** 1 Center for Reproductive Medicine, Department of Obstetrics and Gynecology, Tongji Hospital, Tongji University School of Medicine, Shanghai 200065, P.R. China; 2 Department of Obstetrics and Gynecology, Central South University Affiliated to Xiang Ya Hospital of Reproductive Center, Changsha, Hunan 410008, P.R. China; 3 Translational Center for Stem Cell Research, Tongji Hospital, Tongji University School of Medicine, Shanghai 200065, P.R. China

**Keywords:** laparoscopy, letrozole, ovulation induction, polycystic ovary syndrome

## Abstract

The aim of the present study was to compare the reproductive outcomes of letrozole and laparoscopic ovarian drilling (LOD) in women with clomiphene citrate (CC)-resistant polycystic ovary syndrome (PCOS). A total of 141 women with CC-resistant PCOS were enrolled and randomly allocated into groups A and B. Group A (n=71) received 2.5 mg letrozole from days 5 to 10 of menses for up to six cycles, and group B (n=70) underwent LOD. A 6-month follow-up was performed. No statistically significant difference was found in the baseline clinical characteristics and the major serum hormone profiles, including luteinizing hormone, follicle-stimulating hormone, estradiol and free testosterone, between the two groups. Women receiving letrozole had a lower rate of spontaneous abortion (6.9 vs. 15.8%) and higher clinical pregnancy (40.8 vs. 27.1%) and live birth (38.0 vs. 22.9%) rates; however, the differences were not statistically significant. Letrozole had superior reproductive outcomes compared with LOD in women with CC-resistant PCOS; therefore, letrozole could be used as the first-line treatment for women with CC-resistant PCOS.

## Introduction

Polycystic ovary syndrome (PCOS) is a common cause of reproductive endocrinopathy in women and is characterized by hyperandrogenism, chronic oligo-anovulation and insulin-resistance (1). Previous studies have suggested that PCOS not only leads to disorders of the reproductive axis and reproductive function, but also contributes to the abnormal metabolism of glucose and aliphatic acid, increasing the risk of endometrial and breast cancers (2,3). For infertile woman with PCOS, clomiphene citrate (CC) remains the first-line treatment; however, 15–40% of women do not resume ovulation following CC treatment, which is defined as CC-resistance (4).

Currently, the most common treatments for CC-resistant PCOS are laparoscopic ovarian drilling (LOD) and gonadotropin treatment. Successful pregnancy outcomes for both treatments have been reported (5). There are, however, disadvantages to LOD, as it requires hospitalization and general anesthesia and may lead to pelvic adhesion and ovarian function decrease, which would hinder any subsequent pregnancies. Due to the high sensitivity of the ovaries to gonadotropin stimulation, treatment with human menopausal gonadotropin or pure follicle-stimulating hormone (FSH) is challenging to control and is individually administered to induce several ovulatory follicles, which incurs a substantial increased risk of multiple pregnancies and ovarian hyperstimulation syndrome (OHSS) (6). In addition, the cost of gonadotropin treatment could add a financial burden to the infertile patient; therefore, a convenient, economic and safe treatment method for CC-resistant PCOS is required (7).

Letrozole (LE) is a potent and selective third-generation aromatase inhibitor (AI), which can effectively and highly selectively block the production of estrogen without disturbing other steroidogenic pathways. LE was first used to treat breast cancer and was found to be superior to the previous gold standard, tamoxifen, and more effective than other AIs. Mitwally and Casper (8,9) introduced LE to the ovulation induction field; since then, numerous investigations into LE-induced ovulation have been performed (10–12). According to the reports, the ovulation rate in women with CC-resistant PCOS is between 54.6 and 84.4%. The aim of the present study was to compare LE with LOD, in order to determine a safer, more efficacious and economical method of treating CC-resistant PCOS.

## Patients and methods

### 

#### Patient selection

The present study followed 141 women attending the Center for Reproductive Medicine of Tongji University (Shanghai, China). The women were diagnosed with PCOS based on the Revised 2003 Consensus Diagnostic Criteria for PCOS (13). This study was approved by Tongji Hospital Research Ethics Committee (Shanghai, China), and all participants provided informed consent prior to inclusion in the trial.

#### Inclusion criteria

The criteria for inclusion in the trial were as follows: Clomiphene resistance, i.e. failure to ovulate following 100 mg CC for 5 days for at least three cycles; patent fallopian tubes, confirmed by hysterosalpingography or hysteroscopic diagnosis; normal semen analysis parameters of the patients' spouses according to the modified criteria of the World Health Organization (14); normal serum prolactin, thyroid stimulating hormone and 17-OH progesterone; no systemic disease; no gonadotropin or other hormonal drug treatment during the preceding 3 months; normal blood count and blood chemistry, including glutamic-pyruvic transaminase, glutamic-oxaloacetic transaminase, urea nitrogen, creatinine, glucose and urine analysis. The semen of the patients' spouses was tested to strengthen the comparibility between the two groups. During the period of treatment, all patients were requested to follow a normal diet and rest regime and to avoid intense physical activities in any form and mental stress and fatigue.

#### Exclusion criteria

The exclusion criteria were as follows: Infertility induced by reasons other than PCOS; uterine cavity lesions or ovarian cyst; >40 years old; body mass index (BMI) >26 kg/m^2^; contraindications to general anesthesia; history of pelvic surgery; other endocrine diseases; or a history of liver or kidney disease.

#### Hormone assays and transvaginal ultrasound

The patients underwent baseline hormone assays for FSH, luteinizing hormone (LH), estradiol (E_2_) and free testosterone on the third day of menses, and the LH/FSH ratio was calculated. At the same time, the ovary volume, antral follicle counts and endometrial thickness were measured by transvaginal ultrasound. For patients with amenorrhea or with irregular cycles, the baseline hormone assays were taken improvisationally.

#### Intervention and follow-up

The women were randomly allocated into the either the LE or LOD group (groups A and B, respectively). No medical leading was made during the decision making process. Once the patients had been allocated to one of the two groups, the treatment was revealed to the investigator; however, the doctor responsible for performing the transvaginal ultrasound follow-up assessment was blinded to the treatment groups.

In group A, 2.5 mg LE oral tablets (Adooq Bioscience, Nanjing, China) were administered on the fifth day of menses and then every day for 5 days. Treatment was repeated for up to six cycles if the patient failed to conceive. In group B, laparoscopy was performed under intravenous general anesthesia (Diprivan; AstraZeneca S.p.A., Rome, Italy) with the patient in a supine position. A 5-mm incision was made in the navel, through which a long sheath punctured into the abdominal cavity, and the inflatable pneumoperitoneum (Guangxi University, Yuannan, China) was placed. Another two 5-mm incisions were made on the right and left lower abdomen and the surgical instruments were inserted into the abdominal cavity. The patient was adjusted into a position with the head high up, the pelvic organs were exposed and a comprehensive exploration of the pelvic organs was made, focusing on the structure and position of the adjacent organs of the bilateral ovaries. Once immobilized, each ovary was cauterized at 4–6 points, each for 4 sec at 40 W, at a depth of 7–8 mm and a diameter of 3–5 mm, using a monopolar electrosurgical needle (Kirgen Co., Shanghai, China), according to the size of each ovary. Following cauterization, a bilateral tubal hydrotubation with methylene blue was performed. During the procedure, small pieces of the ovaries were obtained for pathological analysis. The pelvis was irrigated using physiological saline. Ringer's solution (ZiQi Bioscience, Shanghai, China) plus dexamethasone was added into the abdominal cavity to avoid adhesion. The total duration of the procedure, as well as any intra-operative or post-operative complications, was noted. The patients were followed-up for 6 months after the procedure.

In both groups, hormone levels were monitored every third day of menstruation in each cycle following treatment, and comparisons were made with the baseline data in the second menstruation after LE treatment or LOD surgery. The endometrial thickness and follicle size were monitored on days 10, 12 and 14 of menses and the subsequent surveillance time-point was adjusted according to the individual situation until ovulation. Ovulation frequency and mean follicular diameters were recorded in both groups during the six cycles. When at least one dominant follicle reached a diameter of 18–22 mm, 8,000 IU human chorionic gonadotropin (hCG; ZiQi Bioscience) was injected and natural intercourse was advised for 36–48 h. The serum hCG concentration was measured 14 days after hCG injection. Biochemical pregnancy was considered when hCG was >2.5 mIU/ml in the absence of menstruation, and clinical pregnancy was defined by a fetal heart beat monitored by ultrasound at 6 weeks of gestation. Comparisons of biochemical and clinical pregnancy rates between the two groups were made.

#### Statistical analysis

Data were collected and analyzed using the Statistical Package for Social Sciences software version 21.0 (IBM SPSS, Armonk, NY, USA). The measurement data are presented as the mean ± standard deviation. Proportions were compared using the χ^2^ test. A P-value of <0.01 was considered to indicate a statistically significant difference.

## Results

### 

#### Patient data

Of the 147 patients assessed for eligibility, four did not meet the inclusion criteria and two did not consent to participate. The remaining 141 patients were randomly assigned to group A (LE treatment, n=71) or group B (LOD, n=70). No statistically significant differences were found between the two groups in terms of age, BMI, duration of infertility, ovarian volume, amenorrhea rates or baseline hormone levels, including LH, FSH, LH/FSH, E_2_ and free testosterone. The baseline hormone levels were taken at the third day of menstruation (Table I).

#### Hormonal characteristics

**Figure 1.**
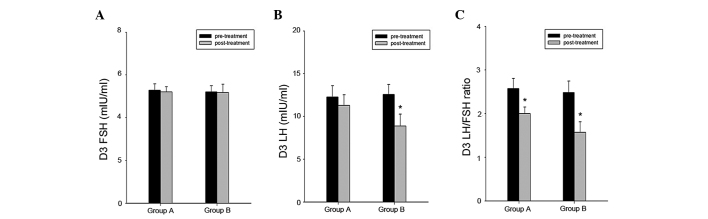
Pre-and post-treatment hormone levels in the two groups. (A) FSH levels, (B) LH levels and (C) LH/FSH ratios. *P<0.01 vs. pre-treatment. FSH, follicle-stimulating hormone; LH, luteinizing hormone; D3, day 3 of the menstrual cycle.

In the second cycle after treatment, the hormone levels were again measured in the two groups (Table II). Compared with the group B patients, the group A patients had a significantly higher LH level (11.30±1.22 vs. 8.89±1.40, P<0.01) and LH/FSH ratio (2.00±0.15 vs. 1.57±0.24, P<0.01). The two groups had a similar level of FSH, E_2_ and free testosterone. When comparing the pre- and post-treatment hormone levels in the two groups (Fig. 1), no clear change in the FSH level was found in either of the groups. There was, however, a marked decrease in the level of LH in the group B. A reduction was also observed in the LH/FSH ratio in the two groups, which was statistically significant in both groups.

#### Reproductive outcomes

**Figure 2.**
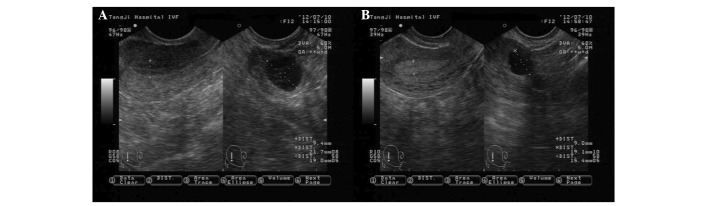
(A) A synchronous cycle. The dominant follicle reached a mean diameter of 17 mm and the endometrium was in the late follicular phase, with increasing hypoechogenic texture between the triple hyperechogenic lines. (B) An unsynchronous cycle with an underdeveloped follicle and a pre-transformed endometrium.

The clinical and reproductive outcomes are presented in Table III. The women were studied for 382 (group A) and 358 (group B) cycles. Ovulation occurred in 305 out of 382 cycles (79.8%) in group A (the LE group) and 237 out of 358 cycles (66.2%) in group B (the LOD group) (P<0.01). In the 305 cycles with ovulation in group A, there were 249 synchronous cycles, in which the endometrium matched with follicle development, which favored implantation; by comparison, 132 cycles out of the 237 in group B were synchronous. The difference between the two groups was statistically significant (81.6 vs. 55.7%, P<0.01). Typical synchronous and non-synchronous images are shown in Fig. 2. The ultrasound images were captured on the day of hCG injection. The endometrial thickness measured on the day of the hCG injection was observed to be significantly increased in group A compared with that in group B (7.82±1.7 vs. 6.21±1.46 mm, P<0.01).

Clinical pregnancy occurred in 29 out of 71 (40.8%) women in group A and 19 out of 70 (27.1%) in group B (P>0.01). Two out of the 29 (6.9%) women in group A had a spontaneous abortion, whereas in group B, 3 out of the 19 (15.8%) women underwent spontaneous abortion; despite the fact that the spontaneous abortion rate was lower in group A, the difference was not statistically significant (P>0.01). The two groups had a comparable live birth rate (38.0 vs. 22.9% in groups A and B, respectively, P=0.05). One case of twin pregnancy was observed in group A and no case of OHSS occurred in either group.

## Discussion

Previous studies (15,16) have suggested that the mechanism by which LE stimulates ovulation may have two parts: The central and the peripheral mechanisms. In the central mechanism, LE acts on the hypothalamus and pituitary in the early follicular phase, and aromatase is then is inhibited. The conversion of testosterone to estrogen is hindered and levels of estrogen in the body are reduced to terminate the negative feedback effect of the hypothalamus or pituitary. FSH is secreted and promotes follicular maturation and ovulation. In the peripheral mechanism, aromatase is a rate-limiting enzyme for testosterone production. LE mainly acts as an AI and prevents the conversion of testosterone to estrogen; testosterone rapidly accumulates in the ovary and FSH receptor gene expression is amplified directly or indirectly; therefore, the follicle is more sensitive to FSH. In addition, testosterone can stimulate insulin-like growth factor, as well as other endocrine and paracrine factors, which promotes the follicular development and ovulation together with FSH (17).

Surgical treatment for PCOS first took place in 1935 (18), when laparotomy ovarian wedge resection was the only way to treat anovulatory PCOS; however, with the development of the retroperitoneal laparoscopic technique, LOD gradually replaced laparotomy ovarian wedge resection. We hypothesize that burning and puncturing the follicle is the main mechanism underlying the efficacy of LOD, as it encourages follicular fluid flow and reduces or eliminates the influence of abnormal hormone and factor levels in the follicle on ovarian function. Furthermore, surgery destroys some of the abnormal structure of the ovary and partially mitigates the abnormal function; therefore, the synthesis of hormones and regulating and growth factors in the ovary is subsequently normalized (19). While the stimulatory and inhibitory interaction between various hormones and factors results in the functionality of the hypothalamus-pituitary-ovarian axis, improvements in the internal ovarian environment can induce normal local control. Patients with PCOS can thus eventually acquire normal ovulatory function (20).

Several studies have been conducted regarding the treatment of CC-resistant PCOS with LE or LOD, and positive results have been achieved; however, there have been few studies comparing the effects of the two treatments (21–23). The present study demonstrated that LE had a superior effect in treating CC-resistant PCOS compared with LOD, and the endometrium was significantly thicker in the LE group than that in the LOD group on the day of hCG injection, which may have resulted from improved angiogenesis of the uterus. In addition, LE has a relatively short half-life of 45 h (CC is 28 days) and, therefore, the lack of estrogen caused by LE would not effect the endometrium and cervix for long (24,25). Furthermore, on the day of hCG injection, ovulation was better synchronized with endometrial development in the LE group, compared with that in the LOD group, which was likely due to LE stimulating more follicles than LOD.

The present study indicated that the LE group had a lower miscarriage rate, although no statistically significant difference was found between the two groups, which have may been due to the small sample size. According to the statistical results, the data showed that the LE group had a better follicle quality, thicker endometrium and more synchronization. In conclusion, compared with LOD treatment, LE treatment is more easily administered and more affordable. In addition, it was shown to improve the ovulation and pregnancy rate of patients with refractory PCOS, especially when combined with LOD. Therefore, LE may be considered a first-line treatment for PCOS.

## Figures and Tables

**Table I.** Baseline clinical and hormonal profiles of the study participants.

Parameter	Group A, n=71	Group B, n=70	P-value
Age^a^, years	29.50±3.26	28.08±3.61	0.707
BMI^a^, kg/m^2^	22.50±1.46	22.41±2.07	0.253
Amenorrhea^b^, n/total n (%)	9/71 (11.2)	12/70 (17.1)	0.456
Years of infertility^a^	3.35±0.43	3.16±0.66	0.120
Volume of ovary^a^, ml	11.47±1.45	12.20±1.11	0.262
D3 LH^a^, mIU/ml	12.25±1.34	12.55±1.17	0.359
D3 FSH^a^, mIU/ml	5.28±0.31	5.21±0.29	0.117
D3 LH/FSH ratio^a^	2.57±0.24	2.48±0.27	0.176
D3 E_2_^a^, pg/ml	50.82±9.49	50.21±9.86	0.323
Hyperandrogenism^b,c^, n/total n (%)	22/71 (20.4)	19/70 (27.1)	0.615

a Values are presented as the mean ± standard deviation

b Values are presented as the number (percentage).

c Hyperandrogenism was defined as free testosterone over the maximum reference value. Group A were administered letrozole treatment; group B underwent laparoscopic ovarian drilling. BMI, body mass index; FSH, follicle-stimulating hormone; LH, luteinizing hormone; D3, day 3 of the menstrual cycle; E_2_, estradiol.

**Table II.** Hormonal characteristics of patients in the second cycle.

Hormone	Group A, n=71	Group B, n=70	P-value
D3 LH^a^, mIU/ml	11.30±1.22	8.89±1.40	<0.001
D3 FSH^a^, mIU/ml	5.20±0.24	5.17±0.40	0.332
D3 LH/FSH ratio^a^	2.00±0.15	1.57±0.24	<0.001
D3 E_2_^a^, pg/ml	50.23±9.86	52.46±9.42	0.271
Hyperandrogenism^b,c^, n/total n (%)	20/71 (28.2)	4/70 (5.7)	<0.001

a Values are presented as the mean ± standard deviation

b Values are presented as the number (percentage).

c Hyperandrogenism was defined as free testosterone over the maximum reference value. Group A were administered letrozole treatment; group B underwent laparoscopic ovarian drilling. FSH, follicle-stimulating hormone; LH, luteinizing hormone; D3, day 3 of the menstrual cycles; E_2_, estradiol.

**Table III.** Reproductive outcomes following treatment in the two groups.

Reproductive outcome	Group A, n=71	Group B, n=70	P-value
Ovulation rate, n/total n (%)	305/382 (79.8)	237/358 (66.2)	<0.001
Endometrial thickness^a^, mm	7.82±1.70	6.21±1.46	0.324
Synchronous cycles, n/total n (%)	249/305 (81.6)	132/237 (55.7)	<0.001
Clinical pregnancy rate, n/total n (%)	29/71 (40.8)	19/70 (27.1)	0.086
Spontaneous abortion rate, n/total n (%)	2/29 (6.9)	3/19 (15.8)	0.372
Live birth rate, n/total n (%)	27/71 (38.0)	16/70 (22.9)	0.050

a Presented as the mean ± standard deviation. Group A were administered letrozole treatment; group B underwent laparoscopic ovarian drilling.
