# [Expression of Concern] Randomized controlled trial comparing letrozole with laparoscopic ovarian drilling in women with clomiphene citrate-resistant polycystic ovary syndrome

**DOI:** 10.3892/etm.2025.13024

**Published:** 2025-11-24

**Authors:** Wei Liu, Shengnan Dong, Yumei Li, Lihong Shi, Wei Zhou, Yingling Liu, Jie Liu, Yazhong Ji

Exp Ther Med 10:1297-1302, 2015; DOI: 10.3892/etm.2015.2690

Following the publication of the above paper, a concern has been raised by an interested reader regarding the statistical handling of the P-values shown in Tables I and II, such that there may be some doubt concerning adequate randomization of the study. The authors have been contacted by the Editorial Office to address the reader’s concern regarding the statistical analysis of certain of the data in this paper; in the meantime, we are issuing an Expression of Concern to notify readers of this potential problem, while the Editorial Office continues to investigate this matter further.

